# Machine Learning Approaches for Assessing Risk Factors of Adrenal Insufficiency in Patients Undergoing Immune Checkpoint Inhibitor Therapy

**DOI:** 10.3390/ph16081097

**Published:** 2023-08-03

**Authors:** Woorim Kim, Young Ah Cho, Kyung Hyun Min, Dong-Chul Kim, Kyung-Eun Lee

**Affiliations:** 1 College of Pharmacy, Kangwon National University, Chuncheon 24341, Republic of Korea; 2 College of Pharmacy, Gyeongsang National University, Jinju 52828, Republic of Korea; 3 The Prime Hospital, 305 Nabulo, Jinju 52828, Republic of Korea; 4 College of Pharmacy, Chungbuk National University, Cheongju 28644, Republic of Korea; 5 Department of Pathology, Gyeongsang National University Hospital, Jinju 52727, Republic of Korea; 6 School of Medicine, Gyeongsang National University, Jinju 52828, Republic of Korea

**Keywords:** immune checkpoint inhibitors, risk scoring, oncology, adrenal insufficiency, machine learning

## Abstract

Adrenal insufficiency is a rare, yet life-threatening immune-related adverse event of immune checkpoint inhibitors (ICIs). This study aimed to establish a risk scoring system for adrenal insufficiency in patients receiving anti-programmed cell death 1 (PD-1) or anti-programmed cell death-ligand 1 (PD-L1) agents. Moreover, several machine learning methods were utilized to predict such complications. This study included 209 ICI-treated patients from July 2015 to February 2021, excluding those with prior adrenal insufficiency, previous steroid therapy, or incomplete data to ensure data integrity. Patients were continuously followed up at Gyeongsang National University Hospital, with morning blood samples taken for basal cortisol level measurements, facilitating a comprehensive analysis of their adrenal insufficiency risk. Using a chi-squared test and logistic regression model, we derived the odds ratio and adjusted odds ratio (AOR) through univariate and multivariable analyses. This study utilized machine learning algorithms, such as decision trees, random forests, support vector machines (SVM), and logistic regression to predict adrenal insufficiency in patients treated with ICIs. The performance of each algorithm was evaluated using metrics like accuracy, sensitivity, specificity, precision, and the area under the receiver operating characteristic curve (AUROC), ensuring rigorous assessment and reproducibility. A risk scoring system was developed from the multivariable and machine learning analyses. In a multivariable analysis, proton pump inhibitors (PPIs) (AOR 4.5), and α-blockers (AOR 6.0) were significant risk factors for adrenal insufficiency after adjusting for confounders. Among the machine learning models, logistic regression and elastic net showed good predictions, with AUROC values of 0.75 (0.61–0.90) and 0.76 (0.64–0.89), respectively. Based on multivariable and machine learning analyses, females (1 point), age ≥ 65 (1 point), PPIs (1 point), α-blockers (2 points), and antipsychotics (3 points) were integrated into the risk scoring system. From the logistic regression curve, patients with 0, 1, 2, 4, 5, and 6 points showed approximately 1.1%, 2.8%, 7.3%, 17.6%, 36.8%, 61.3%, and 81.2% risk for adrenal insufficiency, respectively. The application of our scoring system could prove beneficial in patient assessment and clinical decision-making while administering PD-1/PD-L1 inhibitors.

## 1. Introduction

Immune checkpoint inhibitors (ICIs) have revolutionized cancer treatment approaches, offering novel strategies that have significantly improved the prognosis for patients with various cancer types. The impact of ICIs is clearly demonstrated by the substantial growth in both clinical trials and markets, with an astounding increase of approximately 700% since their initial introduction [1,2]. This remarkable surge signifies the growing recognition and adoption of ICIs as a crucial component of modern cancer therapies. The expanding eligibility for ICIs further emphasizes their widespread adoption and effectiveness. The proportion of cancer patients deemed eligible for ICIs has witnessed a remarkable escalation, rising from 1.5% in 2011 to a staggering 43.6% in 2018 [3]. These numbers underscore the increasing recognition of the potential benefits that ICIs offer in managing and treating cancer. Presently, more than 40% of cancer patients in the United States are receiving ICIs, highlighting the significant impact of this innovative therapeutic approach [4]. Notably, the positive response rates of ICIs in patients with non-small-cell lung cancer (NSCLC) have exhibited promising growth. The proportion of ICI responders among NSCLC patients increased from 5.9% in 2016 to 7.09% in 2018 [3]. Several studies have highlighted the clinical benefits of ICIs in various cancer types. Pembrolizumab showed superior overall response rates and prolonged progression-free survival compared to ipilimumab in advanced melanoma [5]. Additionally, nivolumab exhibited improved overall survival compared to docetaxel in NSCLC patients who had previously received chemotherapy [6]. Atezolizumab also demonstrated significant overall survival improvement and durable responses compared to chemotherapy in patients with locally advanced or metastatic urothelial carcinoma who had prior platinum-based therapy [7]. This trend signifies the growing understanding of the effectiveness and clinical utility of ICIs in managing specific cancer types, providing tangible evidence of their beneficial impact on cancer treatment outcomes.

The increasing utilization and efficacy of ICIs in cancer treatment are testaments to their transformative potential. These immunotherapies have not only improved patient prognosis but have also opened new avenues for personalized and targeted approaches to cancer care. As ongoing research and development continue to enhance our understanding of ICIs and their mechanisms of action, the field holds even greater promise for the future, fostering hope for improved outcomes and quality of life for cancer patients worldwide. Three types of ICIs are approved by the United States Food and Drug Administration; cytotoxic T-lymphocyte-associated protein 4 (CTLA-4), programmed cell death receptor 1 (PD-1), and programmed cell death ligand 1 (PD-L1) inhibitors. CTLA-4 or PD-1/PD-L1 inhibitors increase cytotoxic T-cell activity by T-cell activation and proliferation [8]. Hence, this T-cell reactivation by ICIs results in not only anti-tumor effects but also immune-related complications. CTLA-4 is expressed on the cell surface of CD4-positive and CD8-positive lymphocytes, where it interacts with T-cell co-stimulatory factors presented by antigen-presenting cells (APCs). The binding of CTLA-4 to these co-stimulatory factors leads to a reduction in the production of interleukin 2 (IL-2) and hampers T-cell proliferation. PD-1 is a receptor that is expressed on various immune cell types, such as T cells, B cells, and NK cells. Among its ligands, PD-L1 is found in diverse cell types, including tumor cells, and it actively participates in inhibiting previously activated T cells [9].

The occurrence and severity of immune-related complications linked to immune checkpoint inhibitors (ICIs) exhibit significant variability, contingent upon various factors, such as the specific target, cancer types, and individual patient characteristics. The exact pathophysiological underpinnings of autoimmune-mediated adverse events remain incompletely elucidated. Nevertheless, there is a strong likelihood that the pathogenesis is intricately linked to the mechanism of action exhibited by checkpoint inhibitors. Within the scope of speculation, it is conceivable that genetic predisposition and tumor types might exert a role in contributing to these adverse events [10,11]. Additionally, T-cell-mediated mechanisms are postulated to be the prevailing factors in the development of immune-related adverse events [12]. These complications encompass a broad spectrum, ranging from mild and manageable to severe and even fatal. Notably, the incidence of grade 3 or 4 adverse events in patients receiving ipilimumab, a commonly used ICI, ranged from 5% to 22% [6]. Conversely, patients treated with pembrolizumab, another ICI, demonstrated a lower incidence of such high-grade complications, ranging from 8% to 14% [13]. These statistics underscore the importance of vigilance and careful monitoring of patients undergoing ICI treatment, as well as the need for individualized risk assessment and management strategies.

Despite the increasing utilization of ICIs, the incorporation of predictive features for immune-related adverse events into routine clinical practice remains a challenge. Immune-related adverse events possess the capacity to necessitate the cessation ICI treatment, ultimately leading to treatment failures. Komiya et al. conducted a study where they found that among the patients enrolled, 30% had their therapy discontinued due to immune-related adverse events. Specifically, 22.5% of patients receiving nivolumab and 42.9% of patients receiving pembrolizumab had their treatment halted as a consequence of immune-related adverse events [14]. Moreover, immune-related adverse events can have severe implications, potentially resulting in fatal outcomes for certain patients [15]. Consequently, the identification of predictive factors for immune-related adverse events assumes significant importance. Hence, identifying validated factors that can reliably predict and anticipate these complications is an ongoing area of study and exploration. The intricate interplay among the immune system, cancer biology, and the unique characteristics of individual patients makes it essential to establish comprehensive and validated risk factors that can inform clinical decision-making. By discerning and understanding these predictive features, healthcare professionals can proactively identify patients who are at a higher risk of experiencing immune-related adverse events, enabling tailored interventions and improved patient management strategies.

The development and validation of such predictive factors require rigorous research efforts, including the analysis of large-scale datasets, biomarker identification, and the elucidation of underlying mechanisms. Collaborative initiatives and ongoing studies aim to unravel the complexities surrounding immune-related adverse events, paving the way for the integration of predictive factors into clinical practice. By incorporating these validated factors, healthcare providers can optimize treatment decisions, improve patient outcomes, and minimize the potential risks associated with ICIs. As the field of immuno-oncology continues to advance, it is crucial to prioritize research and development efforts to identify and validate predictive features for immune-related adverse events. This pursuit will not only enhance patient safety and quality of care but also contribute to the wider understanding of immunotherapy mechanisms, further refining the application of ICIs in cancer treatment.

Adrenal insufficiency is one of the immune-related complications associated with ICI treatment and is characterized by a relative deficiency of adrenal cortisol production. It is important to notice ICI-associated adrenal insufficiency as it may result in life-threatening consequences and require lifelong hormone therapy. A study utilizing the World Health Organization’s pharmacovigilance database of individual case safety reports revealed that there was a dramatic increase in ICI-associated adrenal insufficiency reports with the development of new ICI agents [16]. In addition, Salinas et al. showed several clinical cases of adrenal insufficiency secondary to treatment with immunotherapy [17]. Furthermore, a systematic review of 15 case reports concluded that adrenal insufficiency occurred in patients receiving PD-1 inhibitors [18]. Whereas the association between ICIs and adrenal insufficiency has been revealed, the risk factors related to this complication have hardly been identified.

Leveraging the burgeoning applications of machine learning in clinical settings, predictive models have demonstrated efficacy in identifying risk factors associated with complications. Machine learning algorithms are vital in the decision-making process for diagnosing depression from vast, diverse, and often incomplete data gathered from social networks. These techniques offer superior insights compared to traditional methods, enabling early detection and intervention, while also highlighting challenges for future research [19]. For instance, the FastAI technology integrated with the ResNet-32 model improved diagnostic accuracy, potentially leading to early discovery, better management choices, and increased odds of survival, ultimately benefiting patients by reducing complications and death associated with breast cancer [20]. Promisingly, prior studies have successfully employed machine learning techniques to predict the efficacy of immune checkpoint inhibitors (ICIs) in non-small-cell lung cancer (NSCLC) patients [21] and conduct a pan-cancer analysis to forecast responsiveness to ICIs [22]. However, a notable gap exists, as no machine learning-based method has been utilized to predict adrenal insufficiency in patients undergoing ICIs. This underscores the potential and importance of further research in employing machine learning models to enhance risk factor prediction and ultimately improve clinical outcomes.

Risk scoring models, including the HAS-BLED score for major bleeding risk, aid medical professionals in making clinical decisions and providing the best patient care. HAS-BLED score is a practical tool to assess the risk–benefit of patients with atrial fibrillation [23]. Peterseon and Geison showed that the use of the risk score model resulted in a reduction of bleeding risk in patients with anticoagulation treatment [24]. Despite the potential assistance in predicting adrenal insufficiency after ICI administration, the exploration of a risk score model remains unexplored by clinicians. Hence, this study sought to investigate the factors associated with adrenal insufficiency in patients receiving PD-1/PD-L1 inhibitors by employing diverse machine learning models. The ultimate goal was to develop a risk scoring system for adrenal insufficiency induced by ICIs.

## 2. Results

Among the initial cohort of 209 patients enrolled in this study, to enhance the overall reliability of the findings, this study excluded nine patients who had undergone steroid therapy before receiving ICI treatment, thus preserving the integrity of the data. No patients diagnosed with adrenal insufficiency were excluded at any stage, which helped maintain homogeneity among the study participants and minimized potential confounding variables. Consequently, a rigorous analysis was conducted on a final sample size of 200 patients, ensuring a robust and representative dataset. As depicted in Table 1, a noteworthy majority of the patients, accounting for 158 individuals (79.0%), were male, reflecting the gender distribution within the study population. The median age of the patients was calculated to be 67.3 years, with ages ranging from 41 to 87 years, highlighting the broad age range encompassed by the study participants. Remarkably, approximately 91.5% of the patients included in this study were diagnosed with stage 4 cancer, indicating that the majority of the participants were facing an advanced stage of cancer at the time of enrollment. Within the cohort, the administration of various ICIs was observed, including pembrolizumab, nivolumab, and atezolizumab. Notably, a subset of 14 patients (7.0%) experienced a decrease in cortisol levels subsequent to the administration of ICIs. This finding highlights the potential impact of these immunotherapeutic agents on the adrenal glands and emphasizes the need to understand and predict the occurrence of adrenal insufficiency in this specific patient population. In addition, as shown in Table 1, patients taking antipsychotics, proton pump inhibitors (PPIs), or α-blockers showed more adverse events than those without these medications (*p* = 0.03, *p* = 0.02, or *p* = 0.01, respectively).

Multivariable analysis (Table 2) included sex, age, and factors with *p* < 0.05 in univariate analysis (antipsychotics, PPIs, and α-blockers). PPIs (AOR 4.5) and α-blockers (AOR 6.0) were significant risk factors for adrenal insufficiency after adjusting for confounders.

Five features (female, age ≥ 65, antipsychotics, PPIs, and α-blockers) were included in the machine learning models. As shown in Table 3, the average AUROC values of 100 random iterations of five-fold cross-validated models were indicated. The AUROC values for multivariate logistic regression and elastic net indicated acceptable performances of the models; 0.75 (0.61–0.90) and 0.76 (0.64–0.89) [25]. The AUPRC values were 0.48 (0.30–0.65) and 0.51 (0.32–0.70), respectively. Random forest and radial kernel SVM showed sub-optimal performances of the models, with AUROC values of 0.69 and 0.64 (95% CI 0.60–0.83 and 0.46–0.83, respectively) [26]. The AUPRC values for these two models were 0.49 and 0.47, respectively (95% CI 0.31–0.68 and 0.28–0.65, respectively). The details of the hyperparameters used for model training were provided in Table 4.

**Figure 1:**
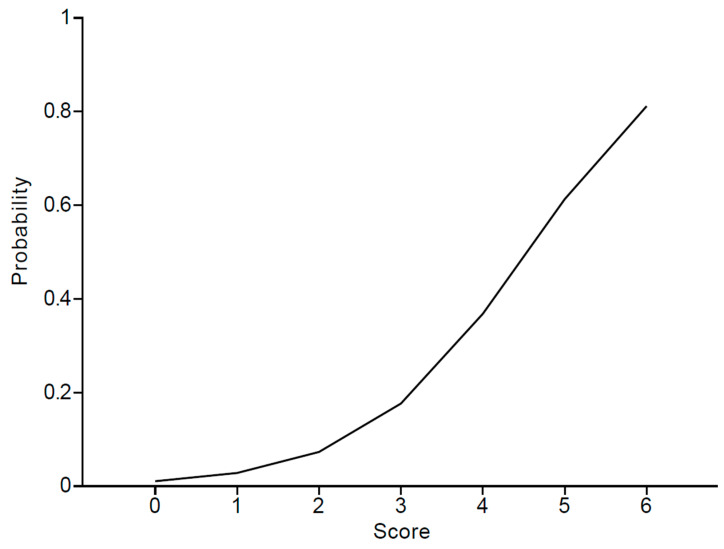
The risk probability according to scores using logistic regression.

As shown in Table 2, for the risk scoring system, female (1 point), age ≥ 65 (1 point), antipsychotics (3 points), PPIs (1 point), and α-blockers (2 points) were integrated into the analysis. Patients with 0, 1, 2, 3, 4, 5, and 6 points showed approximately 0%, 2.3%, 11.1%, 14.3%, 22.2%, 100.0%, and 100.0% risk of adrenal insufficiency, respectively. Figure 1 illustrates the logistic regression curve, which maps the scores to risk scores. Additionally, Table 5 displays the risk probabilities corresponding to the scores obtained through logistic regression. The scoring system achieved an AUROC of 0.80 (95% CI 0.69–0.91).

## 3. Discussion

This is the first study establishing a risk score system for adrenal insufficiency in patients receiving ICI treatment utilizing machine learning methods. A risk score model that incorporated risk factors (female, age 65 and above, antipsychotics, PPIs, and α-blockers) was developed and showed a good prediction rate (AUROC 0.80). From the logistic regression curve, patients with 0, 1, 2, 4, 5, and 6 points showed approximately 1.1%, 2.8%, 7.3%, 17.6%, 36.8%, 61.3%, and 81.2% risk of adrenal insufficiency, respectively. This risk score system could be valuable in clinical practice and patient management, aiding healthcare professionals in identifying high-risk individuals and implementing appropriate interventions to mitigate potential adverse effects of ICI treatment.

PD-1 is known to be highly expressed on tumor-specific T cells, and its transcription is triggered by several transcription factors, such as the nuclear factor of activated T cells and interferon regulatory factor 9 [27,28]. PD-L1 is expressed by tumor cells by an adaptive immune mechanism and is implicated in tumor progression [29,30]. Both PD-1 and PD-L1 are known to negatively regulate T cell-mediated immune responses [31]. The inhibition of PD-1/PD-L1, therefore, results in T cell reactivation and enhances antitumor immunity. For instance, pembrolizumab was shown to roughly double five-year overall survival rates compared to chemotherapy in patients with NSCLC [32]. Moreover, NSCLC patients receiving atezolizumab resulted in longer overall survival than those with chemotherapy by seven months [33].

However, the enhanced anti-tumor effect is accompanied by an increased incidence of immune-related adverse events because of their common pharmacological mechanisms. A systematic review of 125 clinical trials involving 20,128 patients treated with PD-1 or PD-L1 inhibitors revealed an overall incidence of irAEs in 66.0% of cases, with grade 3 or above irAEs in 14.0% [34]. The most common irAEs were skin-related, followed by gastrointestinal toxicity and endocrine-related events [35,36]. Fatal irAEs were relatively uncommon, with cardiac and neurological events being the most prominent causes [37]. In addition, a study showed that pembrolizumab was associated with grade 3 or 4 immune-related adverse events in 14% of patients [38]. Another meta-analysis revealed that approximately 10% of patients with nivolumab displayed endocrine toxicities [39]. Adrenal insufficiency is a potential adverse immune-related outcome of immunotherapy, including ICIs, with the possibility of occurring in primary, secondary, or mixed-type forms. Among these, insufficiency is the prevailing reason for such events [40]. The symptoms often lack specificity and may present as intricate manifestations. The timely exclusion of AI is crucial, and patients should receive close monitoring for AI occurrences even after discontinuing ICI treatment. Additionally, adrenal insufficiency can pose life-threatening risks, and in some cases, may necessitate ongoing glucocorticoid and mineralocorticoid replacement therapy [17]. Thus, it is essential for both healthcare providers and patients to be well-informed about the management of immune checkpoint inhibitor-induced adrenal insufficiency. Thus, awareness of ICI-associated adrenal insufficiency is critical in clinical settings.

This study showed that the use of PPI use was associated with adrenal insufficiency in patients treated with ICIs. PPIs are widely used in cancer patients for the prevention and treatment of gastrointestinal symptoms. Evidence also indicates that they may affect cancer immunity and response to immunotherapy since PPIs can change stomach pH or interact with cytochrome P450 [41]. In addition, PPIs may have pharmacologic interactions by triggering host immune responses, such as direct interaction with major histocompatibility complex proteins [42]. In this context, it can be speculated that the concomitant use of PPIs and ICIs have increased immune-related adverse events. A study revealed that patients receiving PPIs with ICIs had an immune-related adverse event, nephritis [43]. They also demonstrated that ICI- and PPI-mediated nephritis was managed with the discontinuation of omeprazole [43], implicating that the dual usage of PPIs and ICIs may impact the immune state. Given that PPIs use can affect immune responses, their use during ICI treatment could result in various autoimmune complications, including adrenal insufficiency.

Antipsychotics increased the incidence of adrenal insufficiency in patients treated with PD-1/PD-L1 inhibitors in the univariate analysis. Concomitant use of antipsychotics and ICIs is considered a risk factor for immune-related adverse events. Hopkins et al. proposed antipsychotics as potential predictors of ICI toxicity and immune-related complications [44]. Antipsychotics are known to increase the incidence of immune-related adverse events because they decrease cortisol levels. Sonino et al. reported a significant decrease in cortisol levels after the administration of ritanserin [45]. Another study indicated that a healthy population receiving olanzapine resulted in a reduction of cortisol levels [46]. Although an exact mechanism is unknown, it can be thought that the blockade of serotonergic, adrenergic, and histaminergic receptors may have influenced HPA function and cortisol-lowering effects [47,48,49]. Hence, the dual usage of antipsychotics and ICIs may have increased the risk of adrenal insufficiency.

This study also showed that α-blockers were associated with cortisol-lowering effects. Studies have demonstrated decreased levels of cortisol after the administration of α-blockers [50,51]. Laakmann et al. revealed that all basal concentrations of cortisol in patients receiving prazosin were lower than in those without prazosin [50]. Alpha-adrenergic receptors are known to be expressed on various immune cells; alpha-1 adrenergic receptors alter the functions of monocytes, macrophages, and myocytes [52,53]. Moreover, the receptors play vital roles in lymphopoiesis and mast cell degranulation [54]. Given that the use of α-blocker can result in an altered immune function, α-blocker usage during ICI therapy could lead to autoimmune adverse events, such as adrenal insufficiency.

This comprehensive study effectively employed a diverse array of state-of-the-art machine learning approaches, harnessing their immense potential to accurately forecast and detect the presence of adrenal insufficiency in patients who are administered PD-1/PD-L1 inhibitors. The utilization of these sophisticated machine learning methods not only demonstrates a pioneering approach in clinical research but also bestows invaluable benefits to the medical community. Moreover, the intricate construction of a robust risk scoring system represents a significant leap forward, equipping healthcare practitioners with a powerful tool to preemptively foresee, prevent, and effectively manage adrenal insufficiency in individuals undergoing immunotherapy with ICIs. By broadening our understanding and predictive capabilities, this groundbreaking study provides a substantial contribution to the field of adrenal insufficiency research and enhances the standard of care for patients receiving PD-1/PD-L1 inhibitors. In this study, machine learning algorithms, including decision trees, random forests, SVM, logistic regression, and elastic net, were employed to predict adrenal insufficiency risk in patients receiving ICIs. Each algorithm possesses distinct strengths and limitations, which are important to consider in the context of the patient population under investigation. Decision trees are beneficial for their ease of interpretation and ability to capture complex interactions among features, but they may be prone to overfitting. Random forests, as an ensemble method, provide robust predictions by aggregating multiple decision trees, but their interpretability can be compromised due to the ensemble nature [55]. SVM is effective for high-dimensional data and clear class separation, but it might struggle with large datasets and have challenges with interpretability [56]. Logistic regression is straightforward and interpretable, but its performance might be limited when dealing with complex interactions among variables. Elastic net, with its L1 and L2 regularization, handles high-dimensional data well, but its applicability could still be constrained by extremely large datasets [57]. Considering the relatively small sample size and the importance of interpretability in developing a risk scoring system for clinical use, simpler and more interpretable models, like logistic regression and decision trees, seem more applicable to this patient population, while still providing valuable predictive performance to aid in patient assessment and clinical decisions when administering PD-1/PD-L1 inhibitors.

The limitations of this study are that it is a single-centered, retrospective study with a small sample size. For example, in the case of antipsychotics, statistical significance was not obtained in the multivariable analysis because only four patients were receiving the medication. As a result, it is essential to exercise caution when extrapolating the findings of this study to real clinical settings. Moreover, this study lacks a detailed mechanism since it is a clinical study. In addition, this study could not consider the impact of co-medications on cortisol measurements and the performance of the ACTH test. Furthermore, the absence of external validation, which is crucial for assessing the trained model’s performance, should be taken into consideration. Subsequent research should focus on externally validating the current results in clinical settings. Despite these limitations, we believe this study stands as the pioneering investigation into the risk factors linked to adrenal insufficiency in patients treated with ICIs, utilizing artificial intelligence-based machine learning models. As this study developed prediction models based on factors associated with adrenal insufficiency in patients receiving ICIs, these findings offer valuable insights for managing and preventing life-threatening complications. Additionally, these results hold promise for informing the design and development of anti-PD-1/PD-L1 therapy for diverse cancer patients in real clinical settings.

## 4. Materials and Methods

### 4.1. Study Patients and Data Collection

This study consisted of 209 patients who were prescribed ICIs from July 2015 to February 2021. Patients diagnosed with adrenal insufficiency at any point were excluded from the study to ensure a homogeneous cohort and mitigate potential confounding effects on the study outcomes. Moreover, individuals who had received steroid therapy prior to ICI treatment or had incomplete data required for the analysis were also excluded, contributing to data integrity and enhancing the overall reliability of the study. The patients were followed-up continuously at the outpatient clinic of Gyeongsang National University Hospital. Blood samples were taken in the morning for basal cortisol levels. The short Synacthen test, also known as the adrenocorticotropic hormone (ACTH) stimulation test, was performed to identify adrenal insufficiency. It is a diagnostic procedure used to assess the adrenal gland’s response to ACTH stimulation. In this test, a synthetic form of ACTH called Synacthen was injected into the patients, and their serum cortisol levels were measured at baseline and at 30 and 60 min intervals after the injection to evaluate their adrenal function and cortisol secretion dynamics. Data were collected from electronic medical records, and the baseline values of the patient characteristics were recorded at the beginning of ICI prescriptions. The patient baseline data on sex, age, height, weight, smoking history, alcohol history, comorbidities, co-medications, cancer type, cancer stage, and the Eastern Cooperative Oncology Group performance scale (ECOGPS) were collected. In this study, adrenal insufficiency was defined in accordance with the Common Terminology Criteria for Adverse Events (CTCAE), version 5.0 [58], which characterizes it as a disorder resulting from an inadequate production of cortisol by the adrenal cortex, and in some cases, aldosterone. The CTCAE further specifies that grade 2 adrenal insufficiency exhibits moderate symptoms, necessitating medical intervention. To precisely determine low cortisol levels, a clear cut-off approach was employed, defining low cortisol levels as either basal cortisol levels below 5 μg/dL or 30 or 60 min serum cortisol levels below 18 μg/dL.

The Institutional Review Board of the Gyeongsang National University Hospital approved this study (approval number: GNUH 2019-11-041). Informed consent was waived due to the retrospective nature of the study and the utilization of anonymous clinical data. All procedures conducted involving human participants adhered to the principles of the Declaration of Helsinki.

### 4.2. Statistical Analysis and Machine Learning Methods

The chi-square test or Fisher’s exact test was used to compare categorical variables between patients with adrenal insufficiency and those without adrenal insufficiency. Multivariable logistic regression analysis was used to examine the independent risk factors for adrenal insufficiency. Factors with a *p*-value less than 0.05 in the univariate analysis, along with clinically relevant confounders (sex and age), were included in the multivariable analysis. Odds ratios (ORs) and adjusted odds ratios (AORs) were calculated from the univariate and multivariable analyses, respectively. A *p*-value of less than 0.05 was considered statistically significant. Statistical analyses were conducted using IBM SPSS statistics, version 20 software (International Business Machines Corp., New York, NY, USA).

In this study, a diverse range of advanced machine learning algorithms was employed to analyze and predict adrenal insufficiency in patients receiving ICI treatment. These algorithms included multivariate logistic regression, elastic net, random forest, and support vector machine (SVM). Each algorithm played a unique role in uncovering valuable insights and patterns within the data. Multivariate logistic regression is a statistical technique that assesses the relationship between multiple independent variables and a binary outcome. By considering various risk factors and clinical confounders, such as age and sex, this algorithm helped identify significant predictors of adrenal insufficiency. The elastic net algorithm combines the strengths of both L1 and L2 regularization methods, allowing for feature selection and variable shrinkage. It aids in identifying the most relevant risk factors while mitigating potential overfitting issues. Random forest, on the other hand, is an ensemble learning method that constructs a multitude of decision trees and combines their predictions. By leveraging this algorithm, the study could capture complex interactions and nonlinear relationships among risk factors, leading to more accurate predictions. In addition, support vector machine (SVM) is a powerful algorithm for both classification and regression tasks. It seeks to find an optimal hyperplane that separates data points into different classes. By utilizing SVM, the study aimed to uncover distinct patterns and boundaries that differentiate patients with adrenal insufficiency from those without.

To evaluate the performance of these algorithms, several metrics were employed. The area under the receiver–operating curve (AUROC) measures the ability of the models to discriminate between patients with and without adrenal insufficiency. The area under the precision–recall curve (AUPRC) provides insights into the trade-off between precision and recall. Additionally, confidence intervals (CI) were calculated to assess the uncertainty associated with these performance measures. All the methods were implemented with the caret R package. Machine learning analyses were performed using R software version 3.6.0 (R Foundation for Statistical Computing, Vienna, Austria). By employing this diverse array of machine learning algorithms and evaluating their performance using established metrics, this study ensured a robust and comprehensive analysis of the risk factors associated with adrenal insufficiency in patients undergoing ICI treatment. Furthermore, to ensure the generalizability of the model’s performance, a rigorous five-fold cross-validation technique was implemented. The complete dataset was randomly partitioned into five subsets, with each subset serving as a test set, while the remaining four subsets were utilized for training the machine learning models. This process of internal validation was repeated 100 times to accurately assess the prediction power of each model. By repeatedly shuffling the data and conducting cross-validation, the study accounted for potential variations and obtained a robust estimation of the models’ effectiveness in real-world scenarios.

Based on the multivariable and machine learning analyses, a comprehensive risk scoring system was developed. This system aimed to provide clinicians with a practical tool for assessing the risk of adrenal insufficiency in patients receiving ICI treatment. Each coefficient obtained from the logistic regression model was carefully normalized by dividing it by the smallest coefficient value. This normalization process ensured that the relative importance of each risk factor was accurately represented in the scoring system. Furthermore, to simplify the application of the risk score, the normalized coefficients were rounded to the nearest integer, allowing for a straightforward and practical interpretation of the risk scoring system.

## 5. Conclusions

In conclusion: this study represents a pioneering effort in investigating the risk factors associated with adrenal insufficiency in patients undergoing ICI treatment, utilizing advanced artificial intelligence-based machine learning models. To the best of our knowledge, this is the first study to develop prediction models based on factors linked to adrenal insufficiency in ICI-treated patients. The insights gained from our research have the potential to significantly enhance the management and prevention of this life-threatening complication. The predictive models developed in this study offer valuable tools for clinicians to identify high-risk patients and implement timely interventions, ultimately improving patient outcomes and safety during ICI therapy. By leveraging artificial intelligence and machine learning, our findings open new avenues for designing and developing anti-PD-1/PD-L1 therapy in the context of various cancer patients.

## Figures and Tables

**Table 1:** Characteristics of patients in this study.

		Complication	No Complication	*p*-Value
		(n = 14)	(n = 186)	
Sex				0.500
	Male	10 (71.4)	148 (79.6)	
	Female	4 (28.6)	38 (20.4)	
Age ≥ 65 years				0.570
	Yes	10 (71.4)	111 (59.7)	
	No	4 (28.6)	75 (40.3)	
Body mass index ≥ 25 kg/m^2^				0.770
	Yes	6 (46.2)	69 (39.7)	
	No	7 (53.8)	105 (60.3)	
Smoking history				1.000
	Yes	2 (14.3)	37 (19.9)	
	No	12 (85.7)	149 (80.1)	
Alcohol history				1.000
	Yes	1 (7.1)	16 (8.6)	
	No	13 (92.9)	170 (91.4)	
Comorbidities				
Angina				1.000
	Yes	0 (0)	1 (0.5)	
	No	14 (100)	185 (99.5)	
Asthma				0.140
	Yes	1 (7.1)	1 (0.5)	
	No	13 (92.9)	185 (99.5)	
Bronchiolitis				0.140
	Yes	1 (7.1)	1 (0.5)	
	No	13 (92.9)	185 (99.5)	
Buerger’s disease				1.000
	Yes	0 (0)	1 (0.5)	
	No	14 (100)	185 (99.5)	
COPD				0.130
	Yes	3 (21.4)	16 (8.6)	
	No	11 (78.6)	170 (91.4)	
Crohn’s disease				1.000
	Yes	0 (0)	1 (0.5)	
	No	14 (100)	185 (99.5)	
Diabetes mellitus				0.760
	Yes	4 (28.6)	47 (25.3)	
	No	10 (71.4)	139 (74.7)	
Gout				1.000
	Yes	0 (0)	4 (2.2)	
	No	14 (100)	182 (97.8)	
Heart Disease				0.050
	Yes	3 (21.4)	10 (5.4)	
	No	11 (78.6)	176 (94.6)	
Hepatitis B				1.000
	Yes	0 (0)	4 (2.2)	
	No	14 (100)	182 (97.8)	
Hepatitis C				1.000
	Yes	0 (0)	2 (1.1)	
	No	14 (100)	184 (98.9)	
Human immunodeficiency virus				1.000
	Yes	0 (0)	2 (1.1)	
	No	14 (100)	184 (98.9)	
Hyperlipidemia				0.370
	Yes	0 (0)	21 (11.3)	
	No	14 (100)	165 (88.7)	
Hypertension				0.390
	Yes	7 (50)	66 (35.5)	
	No	7 (50)	120 (64.5)	
Hypo-, hyperthyroidism				1.000
	Yes	1 (7.1)	15 (8.1)	
	No	13 (92.9)	171 (91.9)	
Myasthenia gravis				1.000
	Yes	0 (0)	1 (0.5)	
	No	14 (100)	185 (99.5)	
Myocardial infarction				1.000
	Yes	0 (0)	4 (2.2)	
	No	14 (100)	182 (97.8)	
Osteoporosis				1.000
	Yes	0 (0)	2 (1.1)	
	No	14 (100)	184 (98.9)	
Parkinson’s disease				1.000
	Yes	0 (0)	1 (0.5)	
	No	14 (100)	185 (99.5)	
Renal failure				1.000
	Yes	0 (0)	5 (2.7)	
	No	14 (100)	181 (97.3)	
Tuberculosis				1.000
	Yes	0 (0)	1 (0.5)	
	No	14 (100)	185 (99.5)	
Co-medications				
5-HT₃ Antagonists				0.630
	Yes	2 (14.3)	17 (9.1)	
	No	12 (85.7)	169 (90.9)	
5-HT₄ agonists				1.000
	Yes	0 (0)	3 (1.6)	
	No	14 (100)	183 (98.4)	
5α-Reductase inhibitors				0.230
	Yes	2 (14.3)	11 (5.9)	
	No	12 (85.7)	175 (94.1)	
ACE inhibitors/ARBs				1.000
	Yes	1 (7.1)	14 (7.5)	
	No	13 (92.9)	172 (92.5)	
Antibiotics				0.210
	Yes	3 (21.4)	20 (10.8)	
	No	11 (78.6)	166 (89.2)	
Anticoagulants				0.100
	Yes	4 (28.6)	23 (12.4)	
	No	10 (71.4)	163 (87.6)	
Antiepileptics				1.000
	Yes	0 (0)	1 (0.5)	
	No	14 (100)	185 (99.5)	
Antihistamines				0.610
	Yes	0 (0)	15 (8.1)	
	No	14 (100)	171 (91.9)	
Antipsychotics				0.030
	Yes	2 (14.3)	2 (1.1)	
	No	12 (85.7)	184 (98.9)	
Antiviral				1.000
	Yes	0 (0)	3 (1.6)	
	No	14 (100)	183 (98.4)	
Aspirin				0.250
	Yes	1 (7.1)	3 (1.6)	
	No	13 (92.9)	183 (98.4)	
Benzodiazepines				0.060
	Yes	4 (28.6)	19 (10.2)	
	No	10 (71.4)	167 (89.8)	
D2 antagonists				1.000
	Yes	0 (0)	2 (1.1)	
	No	14 (100)	184 (98.9)	
Diuretics				0.230
	Yes	2 (14.3)	11 (5.9)	
	No	12 (85.7)	175 (94.1)	
Dopamine				1.000
	Yes	0 (0)	3 (1.6)	
	No	14 (100)	183 (98.4)	
Metformin				0.690
	Yes	2 (14.3)	23 (12.4)	
	No	12 (85.7)	163 (87.6)	
NSAIDs				0.470
	Yes	3 (21.4)	29 (15.6)	
	No	11 (78.6)	157 (84.4)	
Opioids				1.000
	Yes	10 (71.4)	128 (68.8)	
	No	4 (28.6)	58 (31.2)	
P2Y12 inhibitors				0.590
	Yes	1 (7.1)	11 (5.9)	
	No	13 (92.9)	175 (94.1)	
PPIs				0.020
	Yes	9 (64.3)	60 (32.3)	
	No	5 (35.7)	126 (67.7)	
SSRIs/SNRIs				0.400
	Yes	1 (7.1)	6 (3.2)	
	No	13 (92.9)	180 (96.8)	
Statins				0.470
	Yes	1 (7.1)	33 (17.7)	
	No	13 (92.9)	153 (82.3)	
Thyroid-related medications				1.000
	Yes	1 (7.1)	12 (6.5)	
	No	13 (92.9)	174 (93.5)	
Tricyclic antidepressant				1.000
	Yes	0 (0)	1 (0.5)	
	No	14 (100)	185 (99.5)	
Zolpidem				1.000
	Yes	0 (0)	3 (1.6)	
	No	14 (100)	183 (98.4)	
α-blockers				0.010
	Yes	5 (35.7)	16 (8.6)	
	No	9 (64.3)	170 (91.4)	
β-blockers				0.450
	Yes	1 (7.1)	7 (3.8)	
	No	13 (92.9)	179 (96.2)	
Cancer type				0.860
	Bladder cancer	1 (7.1)	15 (8.1)	
	Colon cancer	0 (0)	3 (1.6)	
	Hepatocellular cancer	0 (0)	12 (6.5)	
	Lung cancer	10 (71.4)	94 (50.5)	
	Pancreatic cancer	0 (0)	2 (1.1)	
	Rectal cancer	0 (0)	11 (5.9)	
	Stomach	1 (7.1)	8 (4.3)	
	Others	2 (14.3)	40 (21.5)	
Cancer stage				0.290
	2	0 (0)	1 (0.5)	
	3	2 (14.3)	11 (6)	
	4	12 (85.7)	171 (93.4)	
ECOGPS				0.160
	0	0 (0)	1 (0.5)	
	1	9 (64.3)	155 (84.2)	
	2	3 (21.4)	16 (8.7)	
	3	2 (14.3)	12 (6.5)	

COPD: Chronic obstructive pulmonary disease; NSAIDs: Non-steroidal anti-inflammatory drugs; ACE inhibitors/ARBs: angiotensin-converting enzyme inhibitors/angiotensin receptor blockers; SSRIs/SNRIs: selective serotonin reuptake inhibitors/serotonin and norepinephrine reuptake inhibitors; ECOGPS: Eastern Cooperative Oncology Group performance scale.

**Table 2:** Univariate and multivariate analyses to identify predictors of adrenal insufficiency in patients receiving immune checkpoint inhibitors.

Characteristics	Unadjusted OR (95% CI)	Adjusted OR (95% CI)	Risk Score (Pt)
Female	1.16 (0.463–5.240)	3.47 (0.860–13.982)	1
Age ≥ 65 years	1.69 (0.511–5.586)	3.08 (0.675–14.085)	1
Antipsychotics	15.33 (1.984–118.526) *	8.36 (0.805–86.816)	3
PPIs	3.78 (1.214–11.768) *	4.46 (1.306–15.247) *	1
α-blockers	5.90 (1.765–19.743) *	6.03 (1.552–23.446) *	2

OR: odds ratio; CI: confidence interval * *p* < 0.05.

**Table 3:** Comparisons of AUC for logistic regression, elastic net, random forest, and SVM models.

	AUROC (95% CI)	AUPRC (95% CI)
Logistic regression	0.75 (0.605–0.896)	0.48 (0.300–0.654)
Elastic net	0.76 (0.637–0.891)	0.51 (0.323–0.702)
Random forest	0.69 (0.559–0.827)	0.49 (0.314–0.676)
SVM (linear)	0.53 (0.304–0.745)	0.36 (0.209–0.514)
SVM (radial)	0.64 (0.459–0.830)	0.47 (0.282–0.654)

Machine learning used the following variables: sex, age, antipsychotics, PPIs, and α-blockers. AUROC: area under the receiver–operating curve; CI: confidence interval; AUPRC: area under the precision–recall curve; SVM: support vector machine.

**Table 4:** Machine learning model specifics.

Method	Hyperparameter	
	Model Specification and Search Grids	Selected Values
Elastic net	λ: 100 equally spaced values in logarithmic scale between 10^−4^ and 0	λ: 0.129155
	α: 0, 0.2, 0.4, 0.6, 0.8, 1	α: 0
Random forest	mtry: 1–5	mtry: 2
SVM with linear kernel	C: 0, 0.001, 0.005, 0.01, 0.05, 0.1, 0.25, 0.5, 0.75, 1, 1.25, 1.5, 1.75, 2, 5	C: 0.5
SVM with radial kernel	Sigma: 2^−15^, 2^−13^, 2^−11^, 2^−9^, 2^−7^, 2^−5^, 2^−3^, 2^−1^, 2, 2^3^	Sigma: 3.051758 × 10^−5^
	C: 2^−5^, 2^−3^, 2^−1^, 2, 2^3^, 2^5^, 2^7^, 2^9^, 2^11^, 2^13^, 2^15^	C: 0.03125

SVM: Support vector machine.

**Table 5:** Risk of adrenal insufficiency according to scores using logistic regression.

Score	0	1	2	3	4	5	6
Risk probability (%)	1.1	2.8	7.3	17.6	36.8	61.3	81.2

## Data Availability

The datasets generated and/or analyzed during the current study are not publicly available due to privacy and ethical considerations. However, interested parties may request access to the data from the corresponding author.
